# Precision Control of Programmable Actuation of Thermoresponsive Nanocomposite Hydrogels with Multilateral Engineering

**DOI:** 10.3390/ijms23095044

**Published:** 2022-05-02

**Authors:** Jisu Hong, Jiseok Han, Chaenyung Cha

**Affiliations:** Center for Multidimensional Programmable Matter, Department of Materials Science and Engineering, Ulsan National Institute of Science and Technology (UNIST), Ulsan 44919, Korea; hjssalt@unist.ac.kr (J.H.); hanjs0209@unist.ac.kr (J.H.)

**Keywords:** nanocomposite, graphene, hydrogel, actuation, programmable, thermoresponsive

## Abstract

Hydrogels capable of stimuli-responsive deformation are widely explored as intelligent actuators for diverse applications. It is still a significant challenge, however, to “program” these hydrogels to undergo highly specific and extensive shape changes with precision, because the mechanical properties and deformation mechanism of the hydrogels are inherently coupled. Herein, two engineering strategies are simultaneously employed to develop thermoresponsive poly(N-isopropyl acrylamide) (PNIPAm)-based hydrogels capable of programmable actuation. First, PNIPAm is copolymerized with poly(ethylene glycol) diacrylate (PEGDA) with varying molecular weights and concentrations. In addition, graphene oxide (GO) or reduced graphene oxide (rGO) is incorporated to generate nanocomposite hydrogels. These strategies combine to allow the refined control of mechanical and diffusional properties of hydrogels over a broad range, which also directly influences variable thermoresponsive actuation. It is expected that this comprehensive design principle can be applied to a wide range of hydrogels for programmable actuation.

## 1. Introduction

Hydrogels are widely used in a diverse array of applications, such as drug delivery systems and cell culture platforms for biomedical applications and pollutant absorbents for environmental remediation [1,2,3,4,5,6,7]. This is largely due to their highly attractive physicomechanical properties, tunable elasticity, and high water absorbency [8,9]. These properties can be efficiently controlled by changing the type and concentration of the constituting polymers and/or crosslinkers. Generating nanocomposite structures, by incorporating functional nanomaterials as fillers to augment the polymeric network, is also broadly explored not only to control their physicomechanical properties but also to impart novel functionalities, such as optical and electrical conductivities [10,11,12].

More recently, stimuli-responsive polymers have been actively adopted to engineer hydrogels that can undergo specific shape changes in response to variable external stimuli (e.g., temperature, pH, and light) [13,14,15]. These “programmable” and “shape-adaptive” hydrogel actuators are being heavily investigated mainly for their potential applications in emerging technologies, such as microfluidics, artificial muscles, and soft robotics [16,17]. Hydrogel actuators are generally fabricated by integrating two or more hydrogels having contrasting swelling/deswelling behaviors, which results in asymmetric deformation. This shape change can be further controlled by incorporating stimuli-responsive functional groups. For example, thermoresponsive polymers, such as poly(N-isopropyl acrylamide) (PNIPAm), undergo volumetric reduction above a certain temperature, i.e., lower critical solution temperature (LCST), as the chains undergo hydrophobic collapse via isopropyl groups [18,19,20]. The hydrogels consisting of polymers containing carboxylate groups can form strong hydrogen bonding at lower pH due to the protonation of carboxylate [21,22,23]. Shape change can also be induced by light-activated molecular transition, such as polymers with azobenzene capable of cis-trans photoisomerization [24].

It is difficult to control precisely the extent of shape change of hydrogel actuators only with stimuli-responsive functional groups, as it is not feasible to incorporate them in a wide range of concentration with limited aqueous solubility and miscibility with other polymers. Furthermore, stimuli-responsiveness and hydrogel mechanics often combine to present diverse, and often complex, shape change effects. Therefore, the programmable stimuli-responsiveness must be accompanied by a careful manipulation of the mechanics and diffusional properties of the hydrogels themselves. To realize the shape control of a hydrogel actuator, hydrogels with contrasting mechanical and swelling behaviors (mismatch) are generally integrated together. For example, a bilayer hydrogel actuator consisting of swelling and non-swelling hydrogel layers undergoes swelling-induced deformation, bending toward the non-swelling layer. However, if thermoresponsive deswelling moieties such as PNIPAm are incorporated into the swelling hydrogel, the swelling layer deswells to a greater extent than the non-swelling layer, resulting in bending in the opposite direction toward the deswelling layer. Another confounding factor is the decrease in mechanical strength commonly demonstrated for more swellable hydrogels intended for greater shape change, which often leads to structural weakness.

**Figure 1:**
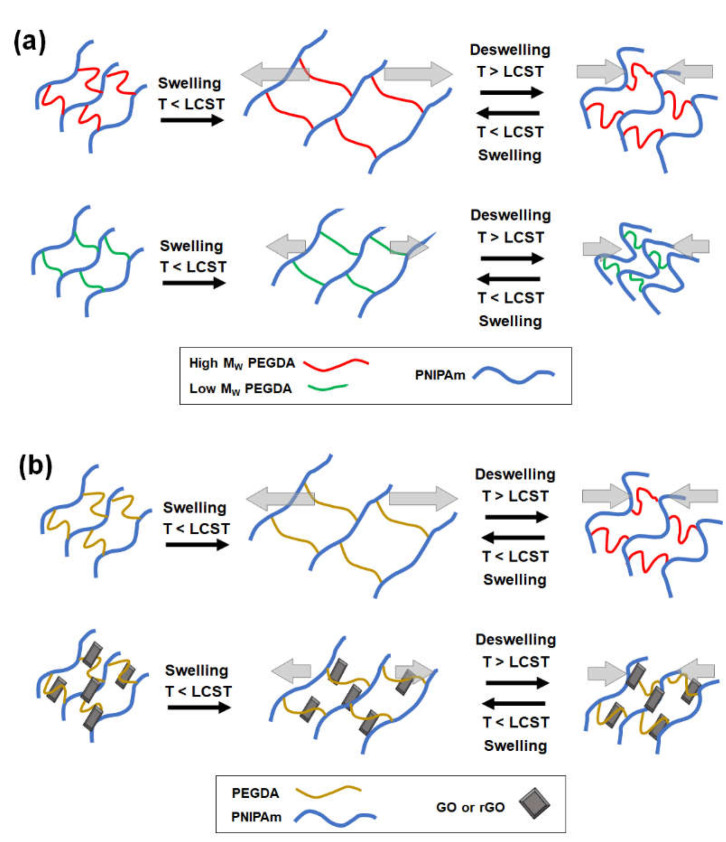
Schematic illustrations of controlling the thermoresponsive PNIPAm hydrogel actuators by (**a**) copolymerizing with PEGDA with varying M_W_ and (**b**) forming nanocomposite with graphene (graphene oxide (GO) or reduced graphene oxide (rGO)). The hydrogels undergo the swelling/deswelling transition around a lower critical solution temperature (LCST).

In order to successfully navigate this complexity of developing a programmable stimuli-responsive hydrogel actuator with precise shape controllability, this study introduces a multilateral approach to control the mechanical and diffusional properties of thermoresponsive PNIPAm-based hydrogels by employing both molecular-scale and nanoscale engineering strategies. First, PNIPAm was copolymerized with poly(ethylene glycol) diacrylate (PEGDA) with varying molecular weights (M_W_) (Figure 1a). Controlling the chain length of PEGDA at comparable concentrations would allow a wide range of shape change of the resulting PEG-PNIPAm hydrogels via variable degrees of swelling [19,25,26]. Second, graphene was incorporated into the PEG-PNIPAm hydrogels to develop composite structures (Figure 1b). Graphene-based nanocomposite hydrogels have been shown to display significant mechanical strength due to strong graphene–polymer interactions [27,28,29,30,31,32]. Therefore, incorporating graphene at a given PEGDA concentration and M_W_ would result in a more subtle control of the physicomechanical properties. In addition, the graphene–polymer interaction was further modulated by incorporating either graphene oxide (GO) or reduced graphene oxide (rGO), which differ in surface energies [33]. Utilizing these graphene-laden PEG-PNIPAm hydrogels, bilayer hydrogel actuators were created, and their programmable deformation was achieved by matching two hydrogel layers with different physicomechanical properties [13,20,34]. Furthermore, since thermoresponsive PNIPAm-based hydrogels are widely explored as a drug release system for expedited release at physiological temperature, the programmable control of drug release of PEG-PNIPAm hydrogel by M_W_ of PEGDA and graphene was also explored.

## 2. Results and Discussion

### 2.1. Physicomechanical Properties of Graphene-Laden PEG-PNIPAm Hydrogels

**Figure 2:**
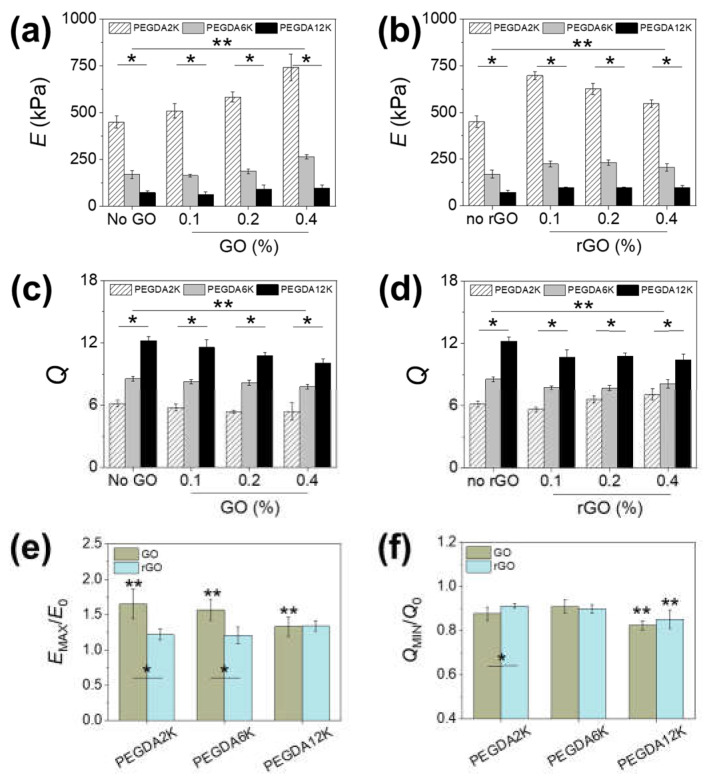
(**a**,**b**) Elastic moduli (*E*) and (**c**,**d**) swelling ratios (*Q*) of PEG-PNIPAm hydrogels with varying concentrations of (**a**,**c**) GO or (**b**,**d**) rGO (* *p* < 0.01 compared between different M_W_ of PEGDA at the same graphene concentration, ** *p* < 0.05 compared between different graphene concentrations at the same M_W_ of PEGDA, *n* = 10). The concentration of PEGDA was 10%. (**e**,**f**) The ratios of the maximum *E* (*E*_MAX_) and minimum *Q* (*Q*_MIN_) by GO or rGO to the *E* and *Q* of pure PEGDA (*E*_0_, *Q*_0_) (* *p* < 0.05 compared between GO and rGO at the same M_W_ of PEGDA, ** *p* < 0.05 compared between different M_W_ of PEGDA, *n* = 10).

The mechanical and swelling properties of PNIPAm-based hydrogels were first controlled by copolymerizing with PEGDA with varying M_W_’s: 2000 Da (PEGDA2K), 6000 Da (PEGDA6K) and 12,000 Da (PEGDA12K). At a given PEGDA concentration, the PEG-PNIPAm hydrogels with higher M_W_ of PEGDA would lead to lower mechanical rigidity and a higher degree of swelling, because of the lower number of acrylic groups and the greater degree of chain relaxation for longer chains [19,25,26]. This explanation was clearly corroborated by the decrease in elastic moduli and the increase in swelling ratio with increasing M_W_ of PEGDA; the moduli ranged from 22 kPa to 213 kPa for 5% PEGDA and from 73 kPa to 450 kPa for 10% PEGDA, and the swelling ratio ranged from 6.7 to 15.5 for 5% PEGDA and from 6.1 to 12.2 for 10% PEGDA (Figure 2 and Figure S1). Obviously, the elastic moduli were higher and swelling ratios were lower at higher PEGDA concentration, but their trends with respect to the M_W_ of PEGDA did not change, which further illustrated the influence of M_W_ of PEGDA in mediating the physicomechanical properties of hydrogels.

Rather than changing the composition and/or concentration of the polymeric network itself, which results in a more substantial change in the overall mechanics, introducing a relatively smaller amount of nanomaterial as a composite filler into the existing polymeric network could allow a more refined control of the hydrogel mechanics. Graphene has been extensively investigated as a nanocomposite filler due to high mechanical strength and favorable physical interaction owing to the unique two-dimensional *sp*^2^ carbon structure [27,28,29,30]. For use in aqueous conditions such as hydrogels, GO having various hydrophilic oxide groups is generally used over unmodified graphene. In addition, GO can be reduced to rGO to eliminate oxide groups and recover *sp*^2^ carbon to varying degrees in order to regain the unique electrical and optical properties and control the hydrophilic–lipophilic balance. To evaluate the effect of a graphene-based nanocomposite system, GO or rGO in various concentrations was incorporated into PEG-PNIPAm hydrogels. At 5% PEGDA, increasing GO up to 0.4% led to 22.5%, 67.2%, and 155.1% increases in moduli for PEGDA2K, PEGDA6K and PEGDA12K, respectively (Figure S1a,e), while at 10% PEGDA there were 65.2%, 56.8%, and 32.8% increases in moduli for PEGDA2K, PEGDA6K, and PEGDA12K (Figure 2a,e). This result clearly proved the mechanical reinforcing effect of GO. Interestingly, the reinforcing effect increased with M_W_ of PEGDA at a lower PEGDA concentration, while it showed the opposite trend at a higher concentration. This contrasting effect of GO could be explained in terms of the different capacity of GO to accommodate physical interaction with polymers. At a higher PEGDA concentration, the inherent cohesion between PEGDA chains was greater than that between PEGDA and GO, so the enhancement in mechanical rigidity was more pronounced at lower M_W_ of PEGDA having higher mechanical rigidity. On the other hand, more PEGDA chains could interact with GO at a lower PEGDA concentration. Since longer chains could interact more closely with GO due to enhanced conformational flexibility, PEGDA with higher M_W_ demonstrated a greater composite effect [29,35,36].

Incorporating rGO showed a similar mechanical reinforcement (Figure 2b and Figure S1b). However, the maximal elastic moduli were demonstrated at intermediate rGO concentrations: 0.1% rGO for PEGDA2K and 0.2% rGO for PEGDA6K and PEGDA12K. This is most likely due to the decreased aqueous dispersibility causing insufficient physical interaction with surrounding polymers at a higher rGO concentration, resulting in a gradual loss in moduli with rGO concentration. The increase in moduli by rGO was generally lower than that by GO regardless of PEGDA concentration, which further attested to the reduced reinforcing effect of rGO (Figure 2e and Figure S1e).

The trends in swelling ratios of GO-linked PEG-PNIPAm hydrogels were inversely proportional to those of elastic moduli, which was largely expected for an elastic network (Figure 2c,d and Figure S1c,d). However, the decrease in swelling ratio by GO or rGO was not as significant as the increase in elastic moduli (Figure 2f and Figure S1f). Since swelling is a consequence of both polymeric chain relaxation and diffusion, introducing a relatively smaller amount of non-extensible nanostructure such as GO or rGO did not contribute greatly to water uptake. Based on these results, it was clearly evident that the mechanical and diffusional properties of PEG-PNIPAm hydrogels could be controlled in a wide range via M_W_ of PEGDA and graphene-based nanocomposite formation.

### 2.2. Thermoresponsive Properties of Graphene-Laden PEG-PNIPAm Hydrogels

#### 2.2.1. Swelling/Deswelling Behavior

**Figure 3:**
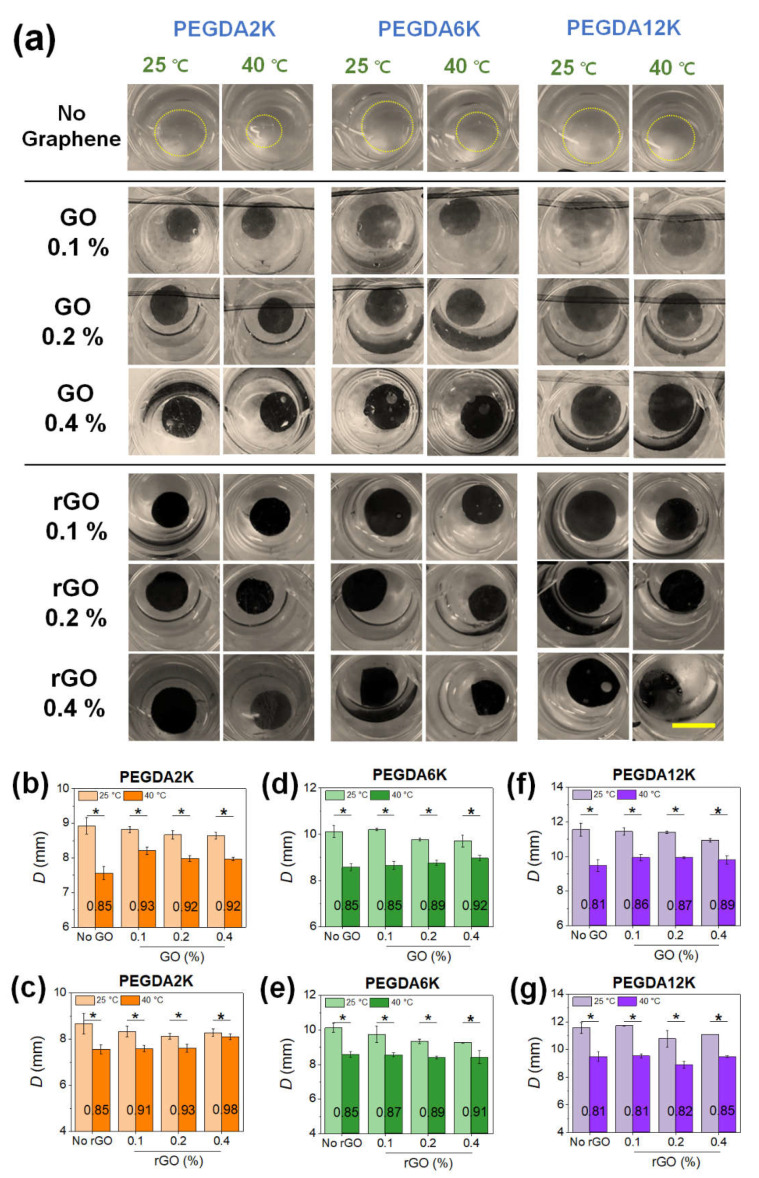
(**a**) Photographs of GO- or rGO-laden PEG-PNIPAm hydrogel disks undergoing thermoresponsive deswelling from 25 °C to 40 °C (scale bar: 1 cm). The concentration of PEGDA was 10%. (**b**–**g**) The diameters (*D*) of the hydrogel disks at 25 °C (*D*_BT_) and at 40 °C (*D*_AT_) shown in (**a**) were quantified: (**b**,**c**) PEGDA2K, (**d**,**e**) PEGDA6K, and (**f**,**g**) PEGDA12K (* *p* < 0.05 compared between 25 °C and 40 °C, *n* = 10). *D*_AT_/*D*_BT_ value at each condition is denoted.

In order to achieve programmable shape transition for hydrogel actuators, thermoresponsive PNIPAm capable of thermoresponsive phase transition is widely utilized. As such, the graphene-laden PEG-PNIPAm hydrogels were also expected to undergo deswelling and concurrent size reduction at a higher temperature. Since the extent of swelling/deswelling of hydrogel is highly dependent on the mechanical properties, the thermoresponsive deswelling behavior would also be highly dependent on the composition of the hydrogels controlled by M_W_ of PEGDA and concentration of graphene. The thermoresponsive deswelling was evaluated by measuring the diameters of hydrogel disks below and above LCST (denoted *D*_BT_ and *D*_AT_, respectively) (Figure 3 and Figure S2).

All hydrogels, regardless of their mechanical properties, underwent size reduction upon increasing temperature, an obvious demonstration of the thermoresponsive effect of PNIPAm (Figure 3a). The size reduction, as evaluated by the ratio of *D*_AT_ to *D*_BT_ (*D*_AT_/*D*_BT_), diminished with increasing concentrations of GO in all M_W_’s of PEGDA, which is a direct consequence of increased mechanical strength resisting deswelling (Figure 3b,d,f). *D*_BT_ increased with increasing M_W_ of PEGDA due to lower mechanical rigidity and higher degree of swelling. *D*_AT_ also increased with M_W_ of PEGDA, since larger hydrogels cannot effectively deswell; this is because it is energetically unfavorable for longer polymeric chains to undergo significant chain collapse due to entropic disadvantage. As a result, the *D*_AT_/*D*_BT_ did not differ significantly by M_W_ of PEGDA at a given GO concentration. Nevertheless, individual *D*_AT_ and *D*_BT_ values could vary widely by GO and M_W_ of PEGDA.

Similar to GO, the inclusion of rGO led to the increase in *D*_AT_/*D*_BT_, owing to increased mechanical strength (Figure 3c,e,g). Unlike GO, however, *D*_AT_/*D*_BT_ was more heavily influenced by M_W_ of PEGDA, where the increase in *D*_AT_/*D*_BT_ with rGO concentration was more pronounced at a lower M_W_ of PEGDA (Figure 3c). At a lower M_W_ of PEGDA, *D*_AT_ increased significantly with rGO concentration, as an increased amount of rGO resists thermoresponsive chain collapse. As shown in Figure 2, increasing rGO concentration resulted in decreased mechanical rigidity and increased swelling due to insufficient physical interaction between more hydrophobic rGO and shorter PEGDA chains. As a result, whereas PEGDA with higher M_W_ having a greater extent of physical interaction with rGO could undergo more facile chain collapse along with rGO leading to lower *D*_AT_/*D*_BT_, PEGDA with lower M_W_ could not undergo more extensive chain collapse leading to higher *D*_AT_/*D*_BT_. Overall, these results illustrate the synergistic effect of copolymerization with PEGDA and nanocomposite formation with graphene on controlling the physicomechanical properties of PNIPAm-based hydrogels, which could ultimately broaden the range of thermoresponsive actuation.

#### 2.2.2. Drug Release Kinetics

**Figure 4:**
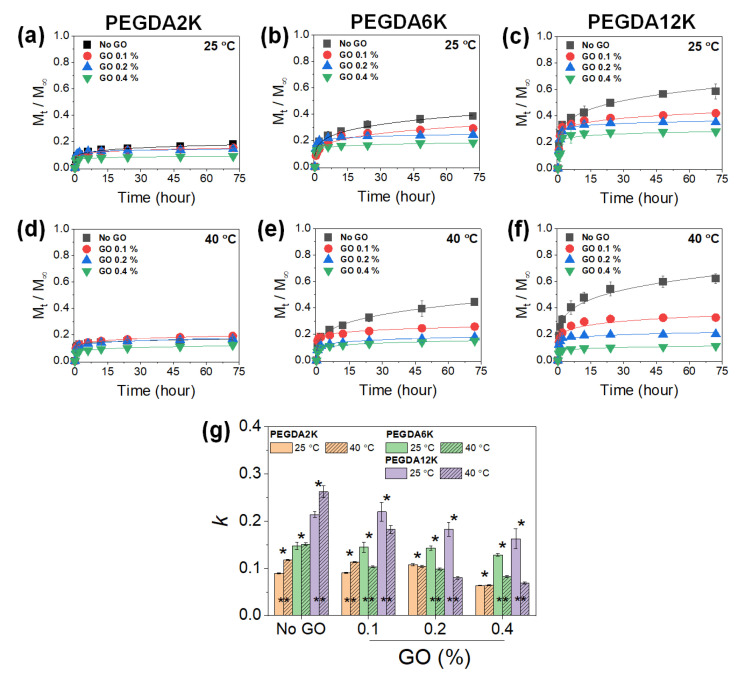
Cumulative release (*M*_t_/*M*_∞_) profiles of BSA released from PEG-PNIPAm hydrogels with varying M_W_ of PEGDA and GO concentration measured at 25 °C or 40 °C: (**a**,**d**) PEGDA2K, (**b**,**e**) PEGDA6K, and (**c**,**f**) PEGDA12K. The concentration of PEGDA was 10%. (**g**) Release kinetic constants (*k*) were obtained by fitting the profiles with Equation (1) (* *p* < 0.05 compared between different M_W_’s of PEGDA, ** *p* < 0.05 compared between 25 °C and 40 °C, *n* = 10).

**Figure 5:**
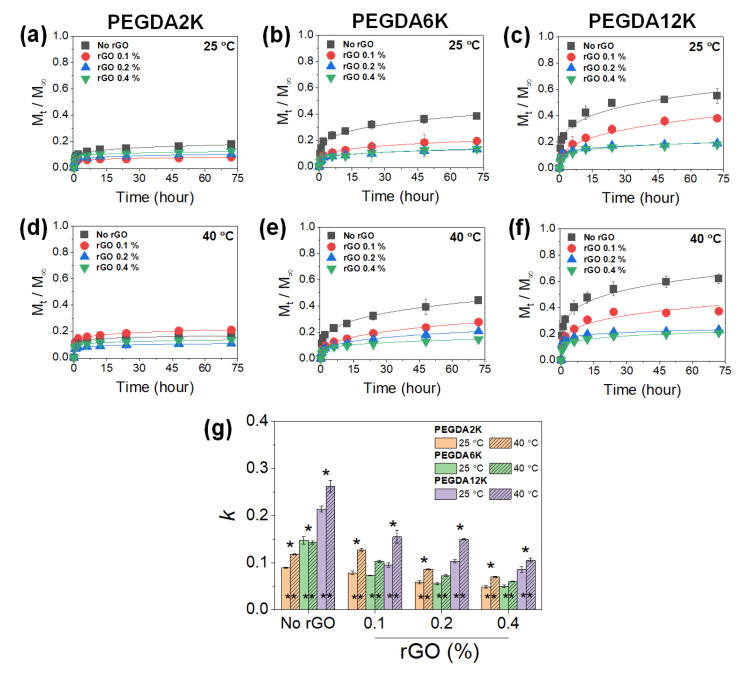
Cumulative release (*M*_t_/*M*_∞_) profiles of BSA released from PEG-PNIPAm hydrogels with varying M_W_ of PEGDA and rGO concentration measured at 25 °C or 40 °C. (**a**,**d**) PEGDA2K, (**b**,**e**) PEGDA6K, and (**c**,**f**) PEGDA12K. The concentration of PEGDA was 10%. (**g**) Release kinetic constants (*k*) obtained by fitting the profiles with Equation (1) (* *p* < 0.05 compared between different M_W_’s of PEGDA, ** *p* < 0.05 compared between 25 °C and 40 °C, *n* = 10).

PNIPAm-based hydrogels have long been studied as a stimuli-responsive drug delivery system, as the drug release from the hydrogels becomes highly facilitated at the physiological temperature. It was thus envisioned that the PEG-PNIPAm hydrogels laden with GO or rGO could be utilized as a programmable drug delivery system that allows refined control of drug release with thermoresponsive actuation. To confirm this hypothesis, bovine serum albumin (BSA) was encapsulated into the hydrogels, and the release kinetics were evaluated by measuring the time-dependent cumulative release profiles, which were then fitted with Equation (1) to obtain release kinetic constants (*k*) (Figure 4, Figure 5, Figures S3 and S4).

At a given GO concentration, *k* increased with the M_W_ of PEGDA, while it decreased with the GO concentration at a given M_W_ of PEGDA, as expected, as a result of diminishing mechanical properties and increasing diffusional properties (Figure 4). Without GO, increasing the temperature resulted in increased *k*, which confirmed the thermoresponsive release by PNIPAm. However, with increasing GO concentration, the thermoresponsive release became opposite, where the release was retarded at a higher temperature, especially at a higher M_W_ of PEGDA. This interesting and counterintuitive result was possibly due to the physical adsorption of BSA on GO. It has been widely demonstrated that various proteins can undergo strong physical adsorption onto the surface of GO [37]. At a higher temperature, the PNIPAm chain collapse via hydrophobic interaction further facilitated the physical adsorption of BSA onto GO, leading to substantially reduced release.

With the inclusion of rGO, *k* values similarly decreased with increasing mechanical properties, increasing the rGO concentration and decreasing the M_W_ of PEGDA (Figure 5). Unlike GO-laden hydrogels, the *k* values increased at a higher temperature at all hydrogel compositions, highlighting the typical thermoresponsive drug release by PNIPAm. This result signified that unlike GO, the physical adsorption between BSA and rGO was greatly subdued. Since proteins require a certain hydrophobic–hydrophilic balance for optimal physical adsorption, rGO was likely too hydrophobic to induce significant physical adsorption of BSA. As a result, more distinctive thermoresponsive BSA release could be demonstrated. Taken together, thermoresponsive drug release could be tuned in a complex manner using PEG-PNIPAm hydrogels incorporated with various amounts of GO or rGO.

### 2.3. Thermoresponsive Actuation of Bilayer Hydrogel Actuators

**Figure 6:**
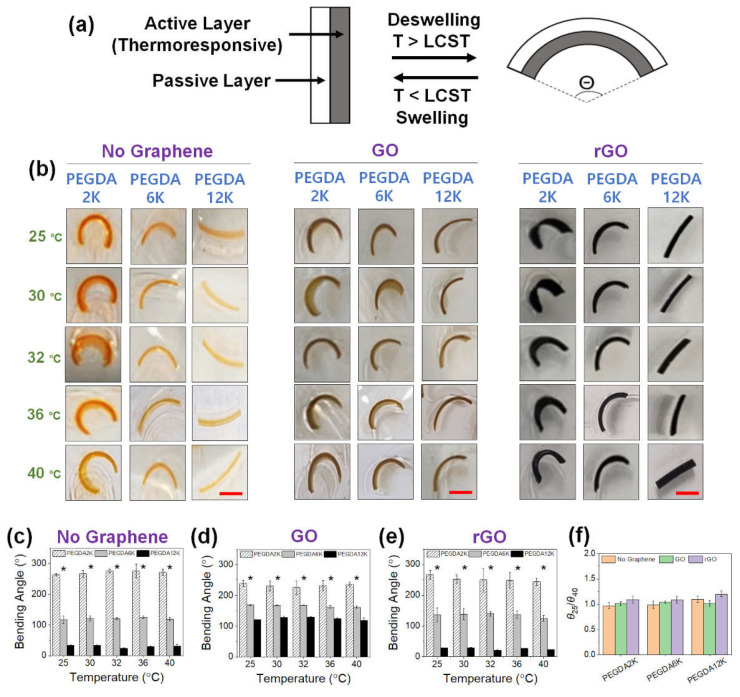
(**a**) Schematic illustration of a thermoresponsive hydrogel bilayer actuator. (**b**) Photographs of GO or rGO-laden PEG hydrogel actuators, without PNIPAm, at various temperatures from 25 °C to 40 °C (scale bar: 5 mm). The concentration of GO or rGO was 0.2%. For PEGDA hydrogels without graphene, orange dye was added for better visualization. (**c**–**e**) The bending angles were quantified from the images in (**b**) (* *p* < 0.05 compared between different M_W_’s of PEGDA, *n* = 10). (**f**) The ratios of bending angles measured at 25 °C (*θ*_25_) and 40 °C (*θ*_40_).

The controllable change of shape of graphene-laden PEG-PNIPAm hydrogel was utilized as a thermoresponsive bilayer actuator. The bilayer actuator consisted of a passive layer, which was fabricated only with PEGDA12K, and an active layer, which was fabricated with PNIPAm, PEGDA, and GO (or rGO) (Figure 6a). The thermoresponsive nature and variable mechanical properties of the active layer would drive the bilayer actuator to undergo the programmable bending phenomenon. To ensure maximal bending, the passive layer was made only with PEGDA12K having the lowest mechanical strength and the highest degree of swelling. While controlling the mechanical properties of the active layer with M_W_ of PEGDA and GO (or rGO), the curvature of the hydrogel actuator was evaluated by measuring the bending angle at various temperatures from 25 °C to 40 °C.

First, the PEGDA hydrogel bilayer without PNIPAm was developed to explore the sole effect of hydrogel mechanics in mediating varying degrees of actuation (Figure 6). Without GO or rGO, the curvature increased with decreasing M_W_ of PEGDA of the active layer due to increasing mechanical mismatch with the passive layer (Figure 6b,c). There was obviously negligible shape change for PEGDA12K having the same polymer composition as the passive layer. With the inclusion of GO, the curvature significantly increased for PEGDA6K and PEGDA12K due to the increased mechanical rigidity by GO (Figure 6b,d). That the increase in curvature for PEGDA12K was especially more enhanced despite having the same polymer composition was indicative of the favorable physical interaction between GO and longer polymeric chains. On the other hand, the curvature slightly decreased for PEGDA2K, which conversely signified the reduced physical interaction with GO causing a small reduction in the mechanical properties.

With the inclusion of rGO, the curvature decreased for PEGDA6K and PEGDA12K as compared with those with GO (Figure 6b,e). Reduced physical interaction with more hydrophobic rGO likely diminished their mechanical strength. However, the curvature for PEGDA2K was larger than that with GO. Since there are more acrylic groups per concentration for PEGDA2K, the increased hydrophobic interaction between rGO and PEGDA2K possibly enhanced mechanical strength, leading to a larger curvature. As expected, without thermoresponsive PNIPAm the effect of temperature on the hydrogel actuation as determined by the ratio of bending angles at 25 °C and 40 °C (*θ*_25_/*θ*_40_) was minimal, and the actuation of the hydrogel bilayer was solely based on the contrasting mechanical properties of the passive and active layers.

**Figure 7:**
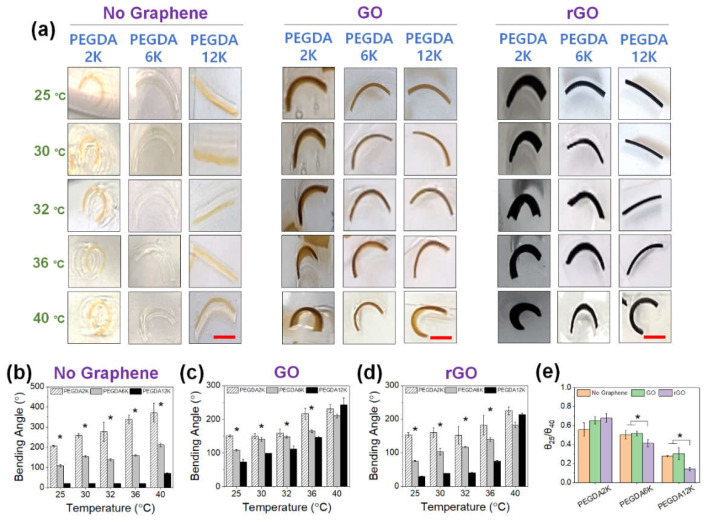
(**a**) Photographs of GO or rGO-laden PEG-PNIPAm hydrogel actuators at various temperatures from 25 °C to 40 °C (scale bar: 5 mm). The concentration of GO or rGO was 0.2%. For PEG-PNIPAm hydrogels without graphene, orange dye was added for better visualization (**b**–**d**) The bending angles were quantified from the images in (**b**) (*p < 0.05 compared between different M_W_’s of PEGDA, *n* = 10). (**e**) The ratios of bending angles measured at 25 °C (θ_25_) and 40 °C (θ_40_).

Next, PEG-PNIPAm hydrogel bilayer was used to explore the combined effects of mechanical properties and thermoresponsiveness on the actuation (Figure 7). The overall trend in curvature was not affected by the presence of PNIPAm, where the curvature was larger for a lower M_W_ of PEGDA (Figure 7a,b). Incorporating GO also similarly increased the curvature for PEGDA12K while reducing the curvature for PEGDA2K and PEGDA6K (Figure 7a,c). In addition, the inclusion of rGO conversely reduced the curvature of PEGDA6K and PEGDA12K as compared with those with GO (Figure 7a,d).

More importantly, due to the presence of PNIPAm, increasing temperature resulted in a substantial increase in curvature, regardless of the hydrogel compositions (Figure 7e). Without graphene, the thermoresponsive increase in curvature was generally gradual with temperature, with the *θ*_25_/*θ*_40_ around 0.6. However, with the inclusion of GO or rGO, the thermoresponsive curvature increased more dramatically, more so for rGO and a higher M_W_ of PEGDA. This highly fascinating result may have been a consequence of increased hydrophobicity by rGO and, to a lesser extent, GO, allowing more extensive hydrophobic interaction with PNIPAm, facilitating more substantial chain collapse. This enhanced hydrophobic effect was compelling enough that the curvature became similar at 40 °C for all M_W_’s of PEGDA. Taken all together, the PEG-PNIPAm hydrogel bilayer could be used as a highly effective programmable actuator, capable of generating a broad range of thermoresponsive actuation, by controlling the mechanical properties by varying M_W_ of PEGDA and generating graphene-based nanocomposites.

## 3. Materials and Methods

### 3.1. Materials

Poly(ethylene glycol) (M_n_ 2000, 6000, 12,000 g mol^−1^) (PEG2K, PEG6K and PEG12K), triethylamine (TEA), dichloromethane (DCM), graphite (flake), sodium citrate, N-isopropyl-amine (NIPAm), ammonium persulfate (APS), N,N,N′,N′-tetramethylethylenediamine (TEMED), sodium citrate and bovine serum albumin (BSA) were purchased from Sigma-Aldrich (St. Louis, MO, USA). Potassium persulfate, phosphorus pentoxide, sulfuric acid (97%), sodium nitrate, potassium permanganate, hydrogen peroxide (30%), and diethyl ether were purchased from Samchun Chemicals Co. (Seoul, Korea). 

### 3.2. Fabrication of Graphene-Laden Thermoresponsive Hydrogel

A hydrogel precursor solution consisting of various formulations of (1) NIPAm, (2) PEGDA, and (3) graphene (GO or rGO) with desired concentration was prepared. The concentration of NIPAm was kept at 10% (*w*/*v*), while the concentration of PEGDA was kept at 5% or 10% (*w*/*v*). The concentration of graphene was controlled up to 0.4% (*w*/*v*). The precursor was sonicated for 1 min to disperse graphene. After adding 20 μL of APS (1 M) and 2 μL of TEMED as radical co-initiators to the 1 mL precursor solution, the solution was immediately placed between two slide glasses with 1 mm spacer, resulting in hydrogel formation. The hydrogel disks (8 mm in diameter) were punched out and incubated in phosphate-buffered saline (PBS, pH 7.4) at room temperature for 24 h before characterization.

### 3.3. Hydrogel Characterization

#### 3.3.1. Mechanical Properties

The mechanical properties of hydrogel were evaluated by measuring elastic moduli from uniaxial compression tests (Model 3343, Instron) [29]. Each hydrogel disk was compressed at a rate of 1 mm min^−1^, and the modulus was calculated as the slope of the stress-strain curve at the initial 10% strain in the elastic region. The swelling ratio was calculated as the weight ratio of swollen hydrogel in PBS at room temperature for 24 h to the dried gel mesh.

#### 3.3.2. Swelling/Deswelling Properties 

The swelling/deswelling properties of hydrogel were evaluated by measuring the change in size at various temperatures. After fully swelling at room temperature, each hydrogel disk was placed in the well of a 24-well plate filled with 1 mL PBS, and the plate was placed in a temperature-controlled incubator. The temperature was gradually increased from 25 °C to 40 °C. After reaching a designated temperature, the plate was kept for 3 h to reach the equilibrium swelling, and the diameter of the hydrogel disk was measured.

### 3.4. Evaluation of Drug Release Kinetics

The drug release characteristics were evaluated by measuring the amounts of BSA as a model protein drug released from the hydrogels. Briefly, 0.5% (*w*/*v*) BSA was dissolved in a precursor solution, and the hydrogel was fabricated as described above. Each hydrogel disk (8 mm) was then incubated in PBS at room temperature or 37 °C. At each designated time, the PBS was collected and the BSA concentration was measured using Pierce^®^ BCA Protein Assay (Thermo Fisher Scientific, Waltham, MA, USA), following the manufacturer’s instructions. The cumulative drug release was plotted versus time and it was fitted using the Ritger-Peppas model,

(1) $$\frac{M_{t}}{M_{\infty }}=k\;\cdot {\;t}^{n}$$

*M*_t_ is the cumulative amount of drug released at a time; *t*, *M*_∞_ is the total amount of BSA in the hydrogel disk; *k* is the kinetic rate constant; and *n* is the exponent related to the release mechanism [38,39,40].

### 3.5. Fabrication and Characterization of Bilayer Hydrogel Actuators

A hydrogel actuator was developed by fabricating two adjoining hydrogel layers with contrasting swelling/deswelling behaviors. One layer (“passive layer”) was fixed with 20% of PEGDA12K, and the other layer (“active layer”) consisted of 10% NIPAm, 10% PEGDA with varying M_W_ (PEGDA2K, PEGDA6K, or PEGDA12K) and graphene (GO or rGO) up to 0.2%. The actuating layer without thermoresponsive properties (devoid of NIPAm) was also prepared as a control, which consisted of 20% PEGDA with varying M_W_ and graphene. The actuating layer (0.5 mm thickness) was first fabricated via radical crosslinking, as described above, which was quickly followed by the passive layer (0.5 mm thickness) fabricated on top of the actuating layer to generate the bilayer structure. The passive layer was fabricated before the actuating layer was fully developed, so the two layers at the interface crosslinked with each other and became irreversibly bonded. The resulting bilayer hydrogel was washed with DI water and then cut into a rectangular strip (5 mm × 15 mm).

The bilayer hydrogel actuator was placed in PBS, and the temperature was controlled from 25 °C to 40 C°. The curvature of the hydrogel actuator was evaluated by measuring the bending angle using Protractor (a free open-source software).

### 3.6. Statistical Analysis

Mean and standard deviation values from ten independent experiments were reported in this study for material characterization (*n* = 10). The statistical significance of differences between two conditions was assessed using the Student’s *t*-test. The statistical significance of difference between multiple conditions was evaluated by one-way ANOVA followed by Tukey’s post hoc test (Microsoft Office Excel). *p* values below 0.05 were considered statistically significant and reported here.

## 4. Conclusions

This study presents a comprehensive strategy for controlling the thermoresponsive actuation of PNIPAm-based hydrogels by modulating their mechanical properties via two approaches. First, PNIPAm was copolymerized with PEGDA with varying M_W_ in order to control their crosslinking density, which allowed the control of mechanical and diffusional properties in a significant range. Increasing M_W_ of PEGDA diminished the mechanical strength and increased the diffusional properties, which led to larger shape deformation. Second, graphene, either GO or rGO, was incorporated into PEG-PNIPAm hydrogels to induce nanocomposite formation, in order to allow more refined and complex control of the mechanics. The polymer–graphene interaction was modulated by the difference in surface properties between GO and rGO. Utilizing these unique programmable capabilities, the graphene-laden PEG-PNIPAm hydrogels were developed into a bilayer actuator, demonstrating a broad range of shape change (curvature). Furthermore, they were shown to be a highly effective drug release system capable of imparting programmable release kinetics displaying varying physicomechanical properties and thermoresponsive actuation. 

## Data Availability

All the data are provided in the manuscript.

## Supplementary Materials

The following supporting information can be downloaded at: https://www.mdpi.com/article/10.3390/ijms23095044/s1.
