# Supplementary material for: Precision Control of Programmable Actuation of Thermoresponsive Nanocomposite Hydrogels with Multilateral Engineering
Source: Int J Mol Sci. 2022 May 2;23(9):5044. doi: 10.3390/ijms23095044 (PMC9103084; doi:10.3390/ijms23095044)
Supplement: Supplementary file 1 [file ijms-23-05044-s001.zip › ijms-1701872-supplementary.pdf]

Supplementary Information

for

Precision control of programmable actuation of thermorespon-sive nanocomposite hydrogels with multilateral engineering

Jisu Hong[1, †], Jiseok Han[1, †], Chaenyung Cha[1,*]

[1]Center for Multidimensional Programmable Matter, Department of Materials Science and Engineering, Ulsan National Institute of Science and Technology (UNIST), Ulsan 44919, Republic of Korea

[*]Corresponding author. Email: ccha@unist.ac.kr (C. Cha)

[†]Equal contribution

1. Experimental

1.1. Synthesis of graphene oxide (GO) using a modified Hummers' method

1.1.1. Pre-oxidation

Graphite (1 g, flake), potassium persulfate (1 g) and phosphorus pentoxide (1 g) were dissolved in concentrated sulfuric acid (10ml) and stirred at 80 °C for 4h. The mixture was cooled down to room temperature, and 200 ml of deionized (DI) water was added slowly. Then, the mixture was centrifuged to collect pre-oxidized graphite, and rinsed with DI water and dried in vacuum oven.

1.1.2. Oxidation

30 ml of sulfuric acid was added to the pre-oxidized graphite and stirred at room temperature. Sodium nitrate (2 g) was added to the mixture while cooling down. Keeping the mixture on ice bath, potassium permanganate (4.5 g) was added slowly. During reaction, the mixture becomes dark green, resulting in thick paste formation. 200 ml of DI water was added slowly while cooling down and stirred manually until it becomes dark red. A hydrogen peroxide (30%) was added slowly until the color of the mixture becomes yellow and no effervescence was detected. Then, the mixture was vacuum-filtered with 0.2μm PTFE membrane to collect the crude GO product. The GO was washed with hydrochloric acid (10%, 200 mL) to remove metal ion and centrifuged (10000 rpm, 30min) for three times. GO is dialyzed against DI water and added DI water for a desired concentration, and it was sonicated for 40 min for dispersion.

1.1.3. Synthesis of reduced graphene oxide (rGO)

rGO was synthesized via GO reduction by using sodium citrate as the reducing agent. In brief, sodium citrate (0.432g) was added to 100ml of GO dispersion (0.36 mg mL^{-1} in DI water) and refluxed at 120°C for 4h. The mixture was centrifuged for 20 min in 10000 rpm to collect rGO

and washed with DI water three times.

1.2. Synthesis of poly(ethylene glycol) diacrylate (PEGDA)

The acrylic functional group was conjugated to the hydroxyl groups of poly(ethylene glycol) via nucleophilic substitution reaction. Briefly, PEG2K (1 equiv.) and TEA (5 equiv.) were dissolved in DCM. Then, acryloyl chloride (4 equiv.) was slowly added and stirred for 24 hours at room temperature under dry N_2. Reacted mixture was filtered to remove insoluble salt and the filtrate was precipitated three times in diethyl ether. Finally, the powder product was dried in vacuum oven overnight to obtain PEGDA2K. The same procedure was followed with the different M_W of PEG (PEG6K and PEG12K) instead of PEG2K for the syntheses of PEGDA6K and PEGDA12K, respectively.

2. Supplementary figures

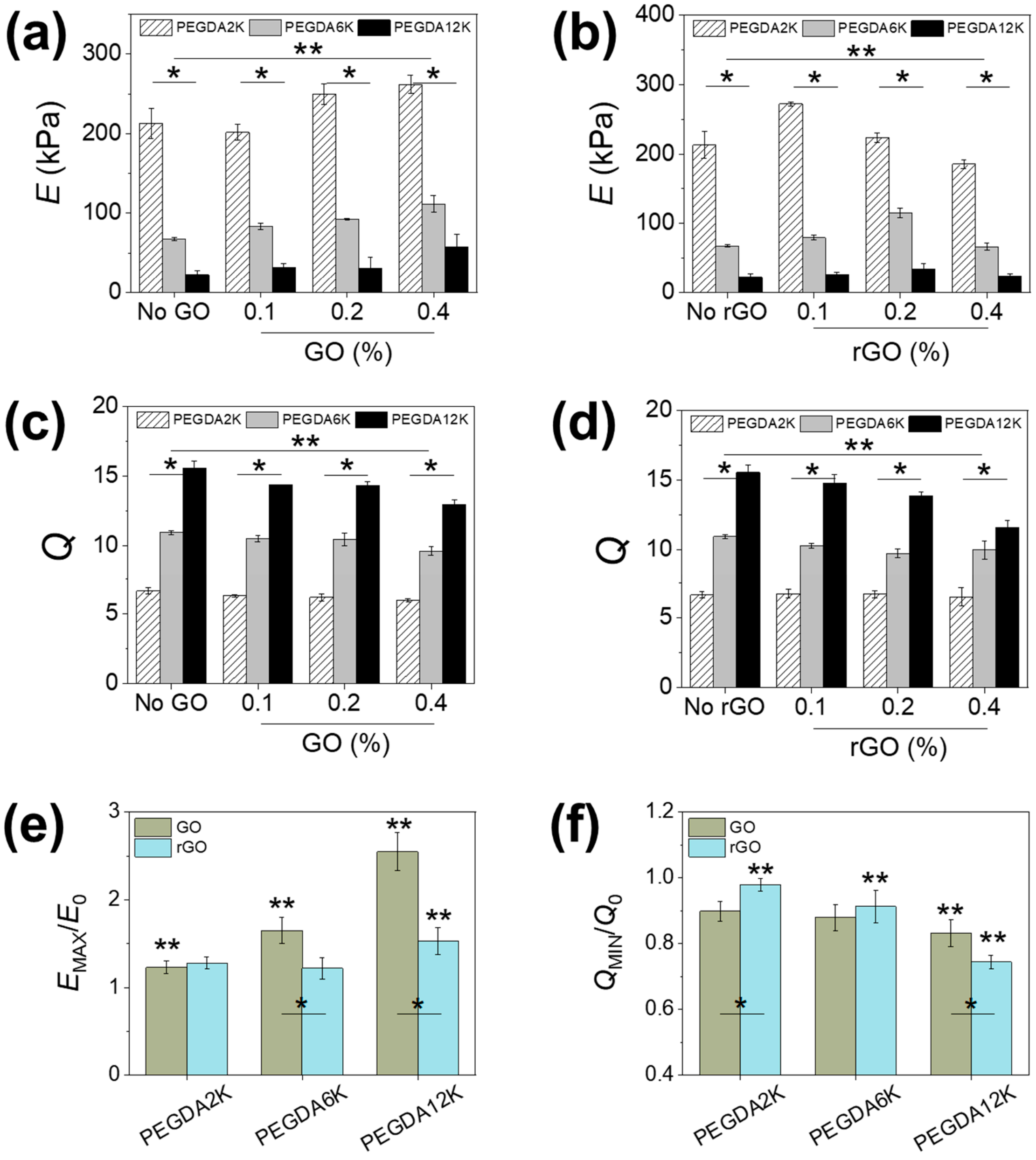

Figure S1. (a, b) Elastic moduli (E) and (c, d) swelling ratios (Q) of PEG-PNIPAm hydrogels with varying concentrations of (a, c) GO or (b, d) rGO (*p<0.01 compared between different M_W of PEGDA at the same graphene concentration, **p<0.05 compared between different graphene concentrations at the same M_W of PEGDA, n=10). The concentration of PEGDA was 5 %. (e, f) The ratio of the maximum E (E_{MAX}) or minimum Q (Q_{MIN}) by GO or rGO to the E and Q of pure PEGDA (E_0, Q_0) (*p<0.05 compared between GO and rGO at the same M_W of PEGDA, **p<0.05 compared between different M_W of PEGDA, n=10).

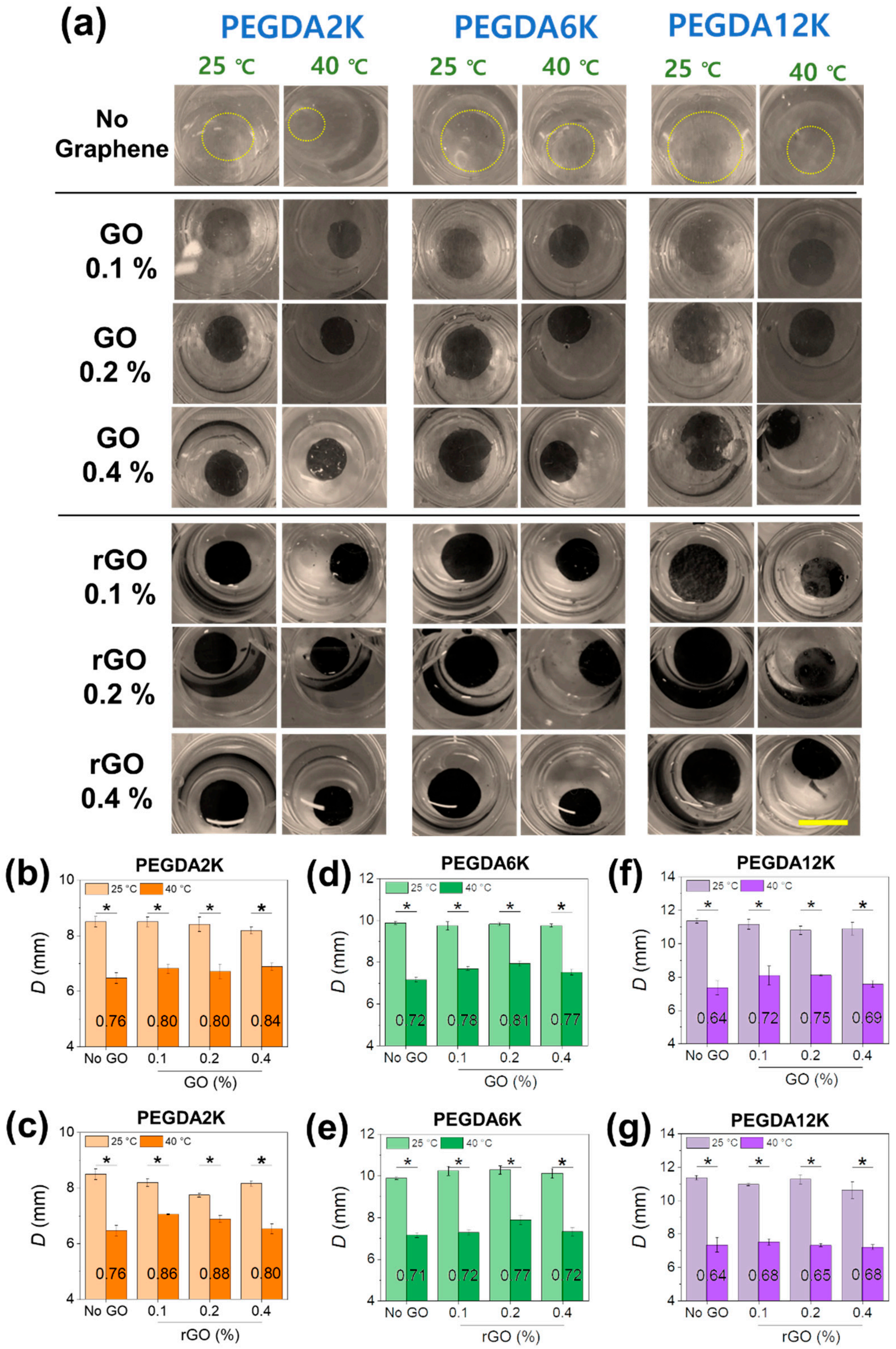

Figure S2. (a) Photographs of GO- or rGO-laden PEG-PNIPAm hydrogel disks undergoing thermoresponsive deswelling from 25 °C to 40 °C (scale bar: 1 cm). The concentration of PEGDA was 5 %. (b-g) The diameters (D) of the hydrogel disks at 25 °C (D_{BT}) and at 40 °C (D_{AT}) shown in (a) were quantified (*p<0.05 compared between 25 °C and 40 °C, n=10). D_{AT}/D_{BT} value at each condition is denoted.

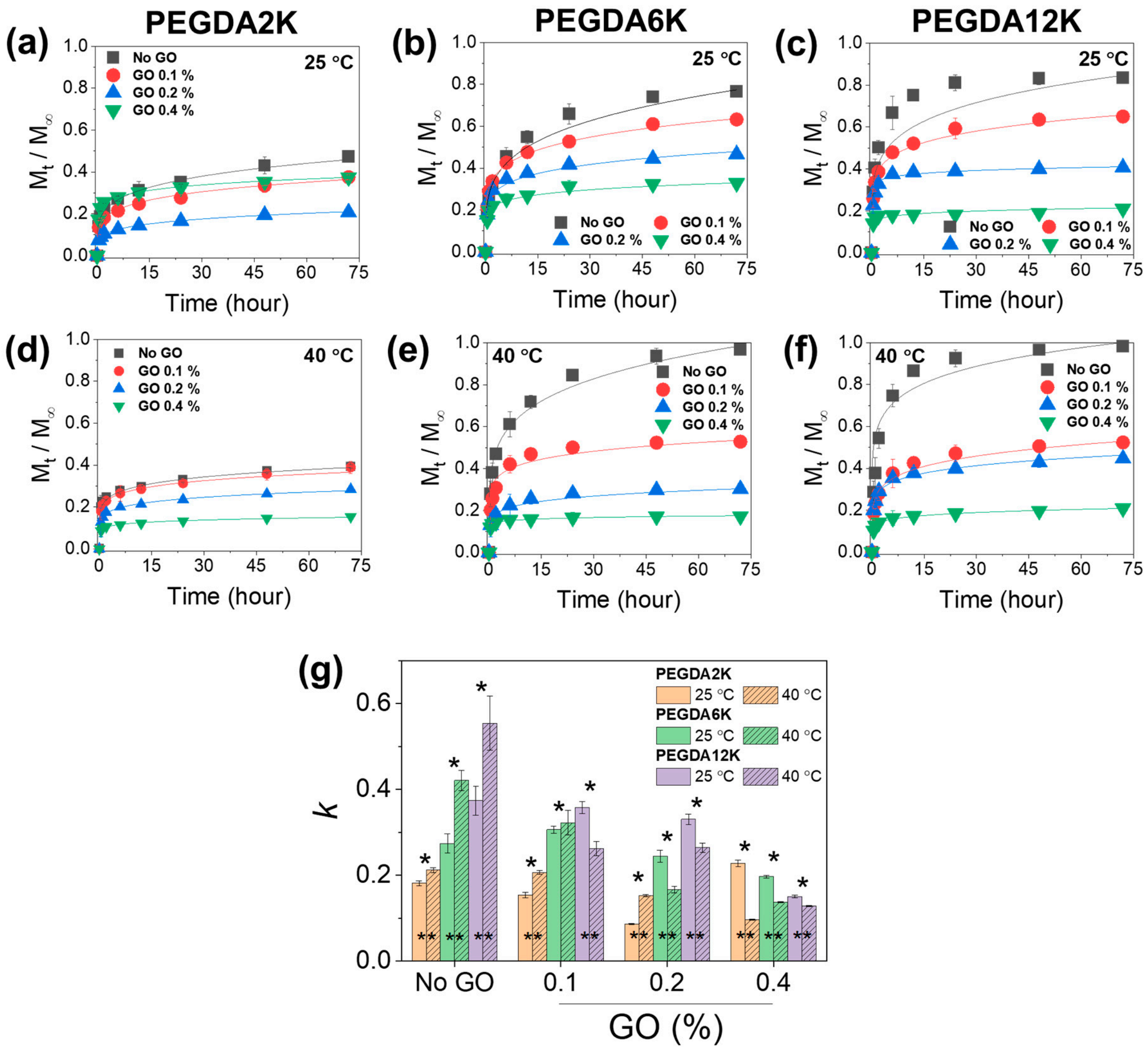

Figure S3. Cumulative release (M_t/M_∞) profiles of BSA released from PEG-PNIPAm hydrogels with varying Mw of PEGDA and GO concentration measured at 25 °C or 40 °C: (a, d) PEGDA2K, (b, e) PEGDA6K, and (c, f) PEGDA12K. The concentration of PEGDA was 5 %. (g) Release kinetic constants (k) were obtained by fitting the profiles with Eq.(1) (*p<0.05 compared between different Mw's of PEGDA, **p<0.05 compared between 25 °C and 40 °C, n=10).

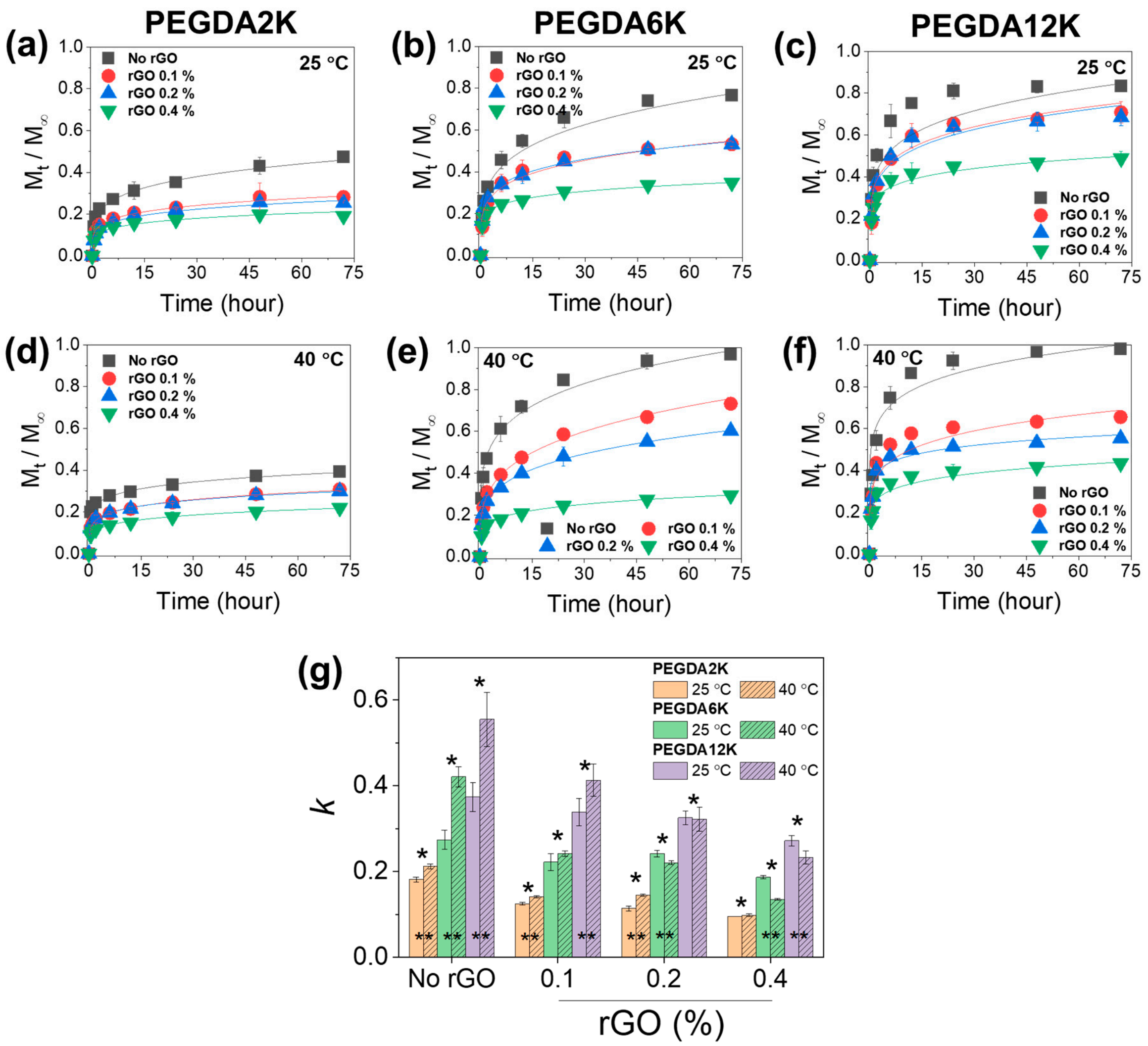

Figure S4. Cumulative release (M_t/M_∞) profiles of BSA released from PEG-PNIPAm hydrogels with varying Mw of PEGDA and rGO concentration measured at 25 °C or 40 °C. (a, d) PEGDA2K, (b, e) PEGDA6K, and (c, f) PEGDA12K. The concentration of PEGDA was 5 %. (g) Release kinetic constants (*k*) obtained by fitting the profiles with Eq.(1) (*$p<0.05$ compared between different Mw's of PEGDA, **$p<0.05$ compared between 25 °C and 40 °C, $n=10$).
